# Hospital and Institutional News

**Published:** 1914-02-21

**Authors:** 


### HOSPITAL AND INSTITUTIONAL NEWS.

### T.R.H. PRINCESS CHRISTIAN AND PRINCESS VICTORIA OF SCHLESWIG-HOLSTEIN.

As we have not failed to insist throughout the St. George's Hospital controversy, and the speech of Mr. Laurence Hardy at the Court of Governors on the 12th inst. demonstrates, H.R.H. Princess Christian, as President of the institution, has not been treated fairly, and was improperly placed in a most unfortunate position. Mr. Hardy insisted that Her Royal Highness was perfectly correct in the attitude she adopted on information supplied to her in her endeavour to look after the interests of the hospital. We rejoice to see that the Governors did their best to do justice to their late President, and to remove the misunderstandings which had arisen by unanimously expressing their regret, and placing on record their unreserved acknowledgment of the right of Princess Christian, in the circumstances described by Mr. Hardy, to institute an inquiry. It is most unfortunate that Princess Victoria of Schleswig-Holstein should have felt it necessary to resign the office of Vice-President. Neither H.R.H. Princess Christian nor Princess Victoria ought to have been placed in the position which caused their resignations. We do but voice the opinion of the hospital world when we say how wide is the sympathy extended to them and how warm is the appreciation of the really valuable and personal services these royal ladies have rendered to the voluntary hospitals during so many years.

### ST. GEORGE'S HOSPITAL.

On another page will be found a report of the special Court of Governors at St. George's Hospital on the 12th instant. Mr. West mentioned some figures which a close examination of the accounts shows to be very misleading. It is satisfactory that our advice to sink all differences and unite in the best interests of St. George's Hospital was adopted by the Governors. An *ex parte* statement was made by Dr. Cahill, a warm supporter of Mr. West. Dr. Cahill's speech was unnecessarily long, it tried the patience of the Governors, and was open to criticism on several points, but it was allowed to pass without comment. Mr. G. R. Turner, the senior surgeon, tersely expounded the views of the medical staff and defined their position in some matters which have given rise to discussion. The Governors unanimously thanked Lord Plymouth and Mr. West, the retiring treasurers, and invited each of these gentlemen to accept the position of a Life Governor of the hospital. Mr. West, in the course of a short speech, expressed his view to be that "the best course I can adopt is to cease entirely to have anything further to do with the affairs of St. George's Hospital." We feel Mr. West's decision is a wise one, and we hope the reconstituted House Committee, with the aid of the new treasurers about to be appointed, may succeed in rapidly bringing the negotiations for amalgamation with the Westminster Hospital to a successful issue. We hope they may further devote their continuous energies to the reorganisation of the financial administration and business management of the affairs of St. George's Hospital, for the position of its finances is serious indeed.

### UNDERSTAFFING AT NORTH EVINGTON INFIRMARY.

In our last issue we referred to an inquest held on an inmate in the above infirmary and to the evidence given that the nursing staff was inadequate in numbers, against which the resident medical officers had appealed in vain. We return to the subject to draw attention to the matron's statement that the staffing of the different wards was left to her and to the committee. If this is the case at North Evington it is in direct contravention of the Orders of the Local Government Board. The Nursing in Workhouses Order, 1897, Art. 5, states: "If in any emergency it appears to the medical officer of the workhouse that the employment of a temporary nurse is required for the proper treatment of any case or cases in the workhouse, and he informs the master of the workhouse in writing accordingly, it shall be the duty of the master to engage a person to act as nurse until the next meeting of the Guardians." A similar provision is contained in the Poor Law Institutions Order, 1913. Again, if the Local Government Board has issued a Special Order for the governance of the North Evington Infirmary similar to those issued to most separate Poor-Law infirmaries, that Order will include as one of the duties of the medical officer "to give the requisite directions as to the treatment, nursing, and diet of the poor persons in the infirmary according to the necessities of their cases." The responsibility rests with the medical officer in charge of the institution, and he cannot delegate his duties in this respect to the matron or to the committee.

### THE MEDICAL OFFICER'S POSITION.

The Guardians certainly have the power at the next meeting to dismiss any nurse employed upon the requisition of the medical officer, but a heavy responsibility would rest upon them if they did so contrary to his advice—a responsibility few Boards would have the temerity to incur. The position of the medical officer would be an unpleasant one in the face of determined opposition from a majority of the Guardians, but it is the duty of a Poor-Law medical officer to meet such opposition unflinchingly.

### ANOCI-ASSOCIATION AT WESTMINSTER HOSPITAL.

An inquest held on February 14 upon a patient who died under anæsthesia at Westminster Hospital has excited some comments in the lay press on account of the special precautions which were taken to render the operation as safe as possible. To those who can read between the lines the report has an added significance, for the principles of "anoci-association" as expounded by Dr. G. W. Crile were put into practice very thoroughly, yet did not avail. The patient was a young woman with a large goitre and very severe symptoms of hyper-thyroidism. It was decided to remove the thyroid gland in the hope of curing the serious symptoms which were being exhibited. Dr. Crile's plan of "stealing" the thyroid was carried out; that is, the patient was not told the date of the operation, but was anæsthetised on four consecutive days with nitrous oxide. Thus she became accustomed to the anæsthetic, and ceased to exhibit any of the symptoms of extreme terror which it at first caused. On the selected day she was once more put under nitrous oxide and oxygen, and the operation done by means of novocain injections, also Crile's invention. Notwithstanding all this the patient died during the operation. It is, of course, unreasonable to condemn a new method on the strength of one case, nor do we imply any such censure; but it is impossible not to take cognisance of these facts in view of the claims made by Crile for his theories and methods, and of the fact that in magazine articles he is being belauded as the equal of Simpson and Lister as a benefactor of humanity. The remaining point of interest is that the anæsthesia was entrusted on all the four occasions to a house physician. In our judgment cases of such exceptional danger should always be anæsthetised by one of the visiting staff anæsthetists, and we think the Westminster Hospital authorities should look into the point.

### THE ABUSE OF NURSES' UNIFORMS.

Dr. Chapple, in the House of Commons on February 12, asked the Home Secretary whether his attention had been called to the instances of the abuse of nurse's uniform during the past few months; and whether he would give his support to a Bill for the State Registration of Nurses, in order to meet this and other evils which were leading to the scarcity of nurses in the ranks of the profession. Mr. McKenna declared that his attention had not been called to any case of the kind during the past few months. The police will make inquiry, but so far as they are aware such cases are of very rare occurrence. Mr. McKenna further pointed out that recent Bills on the subject of the Registration of Nurses have not touched on this matter, and there would be serious difficulties in framing any provisions of the nature suggested. From inquiries we have made we are convinced that a self-respecting nurse in uniform is as safe from insult or molestation in the discharge of her duties, as is the nun in the uniform of her religious sisterhood. Mr. McKenna added that he could not make any promise with regard to a Bill for the Registration of Nurses. The fact is the more this question is discussed, and the more the effects of registration on nursing in other countries are studied, the more statesmen and those responsible for the training and discipline of nurses are convinced that it is in no sense a panacea for existing abuses in nursing, nor is it calculated to greatly advantage the trained nurse as its advocates erroneously preach.

### REGISTRATION OF NURSES.

In our issue last week (pp. 527-8) we dealt with the recent correspondence in the *Times* on this subject. On the 13th inst., after we had gone to press, the following letter appeared from Viscount Knutsford, containing arguments against a Bill: "I am loth just now to enter into public controversy. But I would be grateful if you would allow me to say that if anyone is sufficiently interested in this question to care to know the arguments against the State registering nurses, I shall be happy to send a short pamphlet setting out these arguments. It can easily be shown that State Registration of Nurses would be no protection to the poor nor to the rich; that it would be no guarantee of character, so all-important in a nurse; that it would not be even a guarantee of technical fitness; that it would do harm to nursing generally; that it would cast a slur on the many not fully trained nurses who are doing splendid and devoted work all over the country as village and district nurses; that it would not prevent even criminals from dressing up and pretending to be nurses; that it would be a real danger to the public. The above and other reasons are why the petition against registration was signed by no fewer than 224 matrons of hospitals—and why, if necessary, the signatures of many more could be obtained against it."

### A CONVERSATION THAT PRODUCED £600.

We have alluded before now to the system of Christmas collecting for Gravesend Hospital. A statement has just been issued giving the latest results, which shows that last Christmas 11,613 houses were visited, and that rather more than one-third of the householders responded. The sum total received was £186, and there were eighty-nine collectors, whose average collection was £1 18s. 6¾d. During the three years that this scheme of collecting has been in existence the hospital has benefited from it by considerably over £600. The experience of the hospital is that it is worth undertaking, since an energetic organiser can carry it through with very little expense. There is another point of interest in the scheme, that it originated out of a conversation which the Secretary, Mr. Pearson, had when returning from the British Hospitals Association Conference at Manchester in 1911. This is a good example of the fruitfulness of such conferences, which occasion conversations of every kind to the mutual benefit of the institutions.

### HOSPITAL TREASURER CHARGED WITH FRAUD.

Mr. Benjamin Hoblis, J.P., of Castle Hill, Maidenhead, honorary treasurer of Maidenhead Cottage Hospital, has been committed for trial on a charge of fraudulently converting to his own use sums amounting to some £146 paid to him on behalf of the trustees of the institution. According to the statements made on behalf of the Public Prosecutor, Mr. Hoblis became treasurer in 1907, and in August 1911 sent in a claim to Somerset House for a return of two years' income-tax, which had been taken at the source from the hospital's invested funds, and asked that the amount might be paid to him at his private address. Eventually the claim was in the main approved. On the day in which a receivable order was sent to Mr. Hoblis it was paid into his private account, and no record of its receipt was made in the hospital's ledger. It was important to note, said counsel, that the only evidence of receipts and payments would be such receipt and cash books as Mr. Hoblis would himself put before the auditors. At the general meeting of the subscribers no reference was made to the receipt of this sum. In February 1913 a balance-sheet again was prepared and passed, with again no reference to this receipt by the defendant. Suspicion was first aroused in November last when the defendant was asked to produce the pass-book. The accounts were then gone through and a shortage of over £300 was revealed. He was thereupon asked to resign. A firm of accountants were employed to go through the accounts with Mr. Hoblis, who, it was fair to add, said counsel, gave every assistance. On December 4 last, concluded counsel, the whole of the money owing was paid into the hospital banking account by Mr. Hoblis, and the hospital had not lost anything by his transactions. After evidence had been called, and Mr. Hoblis' counsel had argued that omission to enter items in a book did not come within the purview of the law and that his client was an innocent borrower, believing that he could, as he ultimately did, repay, the magistrates decided, as mentioned above, to commit the defendant for trial at the June Berks Assizes.

### SIR ARTHUR DOWNES ON VENEREAL DISEASE.

As senior medical inspector of the Local Government Board, Sir Arthur Downes gave evidence last week before the Royal Commission on Venereal Diseases. He said that the experience of Poor-Law medical officers, both in London and the provinces, tended to show that venereal diseases were less prevalent among the poor than formerly. He added that while Poor-Law authorities did not insist on venereal cases receiving institutional treatment, yet a considerable proportion discouraged domiciliary treatment for this class of patient, and that he thought this might deter some from seeking it. He paid a striking tribute to the London Poor-Law infirmaries, and said that most of these were able to devote special wards to venereal cases, though the number of these was insufficient to justify such wards being always reserved. The Wassermann tests required by the infirmaries were generally carried out either at the Wassermann Institute or at a clinical laboratory. Laboratories could not be expected in the smaller provincial institutions, but Boards of Guardians had elastic powers to pay for special diagnosis and to send cases to special institutions, whilst their powers of combining for particular purposes were also wide. He could endorse the recommendation of the Poor-Law Commission that, subject to safeguards, the public assistance authority should have power to detain these cases when medically certified as dangerous to others, only if the detention were non-penal and arranged so as to be as little deterrent as possible. The common-sense statesmanship of this view is evident from the fact that it is to the public interest that such cases should not conceal themselves, which they would certainly have the strongest motives for doing if revelation were to mean punishment. We earnestly trust that Sir Arthur Downes' admirable arguments will have the full weight to which they are entitled fully accorded to them in the Commission's report.

### OFFICIAL ADVICE ON CHEAP SANATORIA.

Readers of the debate on Welsh sanatorium construction and equipment which we published last week will be interested to compare the specifications put forward with the advice given in a circular to county councils issued almost simultaneously by the Local Government Board. Prepared by Mr. Brook Kitchin, the architect, and Dr. Newsholme, the medical officer to the Board, the object of the circular is to give hints on the economical provision of sanatoria. The number of 100 beds in buildings and ten in shelters is taken as a unit, and a site of fifty acres is recommended, or, when land is dear, not less than twenty. Recreation rooms are discouraged, and consequently a dining room is recommended as sufficient. Heating is stated to be usually unnecessary in the patients' quarters, except in rooms where special nursing is required, and in the dining room, and a low-pressure system is recommended on economical grounds. The speakers last week were fairly unanimous in thinking heating of patients' rooms to be unnecessary, except Dr. Louis Parkes, who urged its importance where sanatoria were built on exposed sites, and few will be found to contradict the official plea for electric lighting. But all these details do not answer the main question: What is the most economical cost per head of an efficient sanatorium?

### THE FATAL VOGUE OF EUGENICS.

As Professor Karl Pearson pointed out some time ago, eugenics has had the fatal set-back of an advertised vogue, with the result that Sir Francis Galton's pioneer work for race improvement is involved in the danger of being equally distorted. The Eugenics Education Society was therefore well advised to hold on Monday night the first of an annual series of lectures in commemoration of the date of his birth, February 16, 1822. Major Leonard Darwin presided, and described the aim of eugenics to increase the probabilities of future generations being endowed hereditarily with noble qualities. He instanced Galton as a type of these. Sir Francis Darwin delivered a lecture in which he said that Galton's eugenic foundation at University College was a proof of his personal belief in the science. He added another belief of Galton's, that a semi-religious horror of non-eugenic marriage might be developed, which he, the lecturer, thought less easy of accomplishment than Galton. On this point there is this to be said, marriage involving the sacrifice of personal choice has been always a characteristic of mankind, from the time of the elaborate regulations of the totem to the dynastic or class marriages which have always imposed themselves on the wills of royal or aristocratic persons. To-day there is, on the whole, "a semi-religious horror" of marriage between members of two different classes, and that being the case, there seems no reason why this horror should not attach one day to "unsuitable" marriages in the physical sense. Is not the real difficulty that we do not really know what unsuitable marriages are?

### WIRELESS TELEGRAPHY AS A HOBBY.

Hobbies of wide and varied interest have at sundry times been alluded to in The Hospital, but mention has not so far been made of wireless telegraphy. Now we learn that among the numerous amateur wireless experts throughout the country is to be numbered the Mayor of Bath, Dr. Preston King, who is also one of the honorary staff of the Royal United Hospital. Dr. King has a "wireless" station, and a few evenings ago was to be seen assisting another local amateur at a "wireless" lecture and demonstration given at the Widcombe Institute.

### SCHOOL MEALS AND SCHOOL TREATMENT.

A deputation from the Parliamentary Committee of the Trades Union Congress waited on Mr. Pease at the Education Office last week, and submitted among other resolutions one asking for an adequate Treasury grant for medical inspection. Mr. Pease said that he had done his best, and that his Department was pressing local authorities to have the children inspected at least three times during the school period, inspection being followed by treatment where necessary. This treatment is still, however, very unsatisfactorily left between voluntary hospitals—for which it has caused great difficulties, and as a rule brought no adequate recompense—a few clinics, and very often mere recommendations which are never carried out. The treatment of school children, which medical inspection has necessitated, should be a branch to itself; to speak of a school service which confines itself to diagnosis is somewhat Gilbertian. Another aspect of this question was touched on by Mr. Pease in his remarks on school meals. He confessed that the law at present seemed to him anomalous, and that he was anxious not to introduce compulsion, which he believed would be unnecessary once the education authorities grasped that money spent on teaching underfed children was wasted. His concluding remark is worth careful attention: the Government would raise the school age to 15 or 16 to-morrow if public opinion would support them. Were public opinion enlightened enough to do that there would be less delay in making school treatment as regular as school inspection.

### RADIUM AND THE HOME RULE BILL.

At the annual meeting of the Manchester and Salford Hospital for Skin Diseases references were made to a recent benefaction of £2,000 for the purchase of radium, the gift of Alderman Holt. Dr. Wild expressed the hope that a committee which is being formed would, through Mr. Holt's offer, develop into a miniature radium institute, not merely for the treatment of cancer, but also for other cases to which radium is found to be beneficial. He also expressed the hope that Professor Rutherford would be asked to join the committee. At the present time the hospital possesses fifteen milligrammes of radium—valued at current prices at £350. It is clear that with Alderman Holt's gift the hospital will be able to acquire six times what it already holds of this precious substance. Even that would scarcely justify a claim to be regarded as a "miniature radium institute." But the men of Manchester are so loyal to their city and so liberal that further benefactions may enable this ambitious scheme to be carried out. Indeed, from a subsequent announcement in the press we learn that representatives of the Manchester hospitals in conference have appointed a committee to discuss the project of one central fund for radium, and that £3,825 has been promised; this sum probably included Alderman Holt's £2,000. We may further note that radium for Glasgow is to be purchased out of the profits of a threepenny pamphlet, printed at its author's expense, written in defence of the Home Rule Bill!

### INFIRMARY OFFICERS AND MATERNITY BENEFIT.

In our issue of January 17 we referred under the above heading to an illegal resolution passed by the Bethnal Green Board of Guardians forbidding any officer of the Guardians to sign a certificate to enable the mother of an illegitimate child, born in an institution of the Guardians, to receive maternity benefit unless the mother gave a written authority for the payment of the benefit direct to the Guardians. A similar resolution was passed by the Darlington Board of Guardians and has been the subject of a letter written by the Local Government Board. In this letter the Board states: "The powers and duties of the Guardians in regard to the recovery of the cost of maintenance from any person legally liable for the same are not affected by any provision in the National Insurance Act; but having regard to Section 3 of the Act, which renders void any agreement to assign benefits, the Board are of opinion that the Guardians should not endeavour to intercept any benefit payable under the Act, and should not make use of the machinery and requirements under the Act to obtain the money payable as benefit or any part of it." This letter gave rise to a discussion in which the Local Government Board was roundly abused for having written it, and a resolution was finally adopted that the letter "should lie on the table." The position of the medical officer was not referred to in the Board's letter, but, as we pointed out before, it is his duty under the General Order of July 1847 and under the Poor Law Institutions Order of 1913 to "certify in writing on the request of any inmate the sickness of the inmate, or the cause of his attendance on him." The Guardians no doubt have the power to forbid the medical officer to sign the maternity form issued by approved societies, but they cannot prevent him giving the above certificate which is probably sufficient to enable an approved society to pay the benefit.

### THE UNFIT FIND A VINDICATOR.

The scattered utterances of Sir J. Crichton-Browne afford him too many opportunities for every one to be taken full advantage of, but the other day he had some good things to say in answer to Sir W. Ramsay on the text of sanitation. In answer to the oft-repeated question, Does not all this social hygiene merely prolong the lives of the unfit; would it not be better to let them die? he answered emphatically No! Certain kinds of the unfit might be prevented from beginning to live, but if coddling was simply a name for sanitary improvement, then we could not have too much of it. Sir James added that it was a popular error to suppose that sanitary measures interfered with natural selection; it certainly kept weaklings alive; but weaklings had often proved to be human benefactors. There you have the answer to the two thoughtless objections often made against thorough-going reforms. First, if you are to organise civilised life at all you had better, and in fact are eventually forced to, organise it properly. Secondly, all this glib talk about the unfit is exceedingly dangerous, since, except in a very limited sense, we do not know what constitutes a fit human being. Any easily manufactured definition which would exclude, say, Julius Cæsar, Keats, and Walt Whitman is clearly no use to any sensible person, although one was an epileptic, another tuberculous, and the third, on any ordinary interpretation of the term, abnormal. That being the condition of our ignorance at present, the less we hurry into drastic measures the better; and, as institutional workers well know, sanitation must be thorough once it is undertaken at all. On the ground where we have some knowledge we are warned not to use it, and on that where we have virtually none we are urged to act. Could anything be more open to ridicule?

### A REMARKABLE RETROSPECT IN ABERDEEN.

The retirement of Mr. G. M. Cook from the chairmanship of the Royal Infirmary, Aberdeen, marks a wonderful period of growth in the history of the institution which has taken place during the thirteen years that he has held this office. In his speech last week at the annual meeting, when his retirement was announced, he gave a glance back at these developments, and summarised them in a very effective way. In 1900, when he took the chair, there was a debt of £24,665 on the hospital, which was paid off by Lord Mount Stephen. Since that time the endowment has increased by £48,365, and by taking the deficiency and the increased endowment together it amounted to £73,030, and with the munificent sum left by the late Mr. David Allan they would certainly have an increase of £85,000. Moreover, £35,780 has been spent on improvements, including the out-patient department and the new operating theatres. In other words, something like £130,000 has been put into buildings or into funds. That is a truly remarkable testimony not only to Mr. Cook's record as chairman, but to the vigour of the voluntary system in Aberdeen.

### AN INGENIOUS HOSPITAL ADVERTISEMENT.

Those who study regularly the first column of the *Times* cannot have failed to observe the frequency of certain *In Memoriam* notices of the following type: "Blank.—In grateful memory of Blank Blank, who died January 1, 1880, a generous benefactor by will of the National Hospital for the Paralysed and Epileptic (Albany Memorial)." We assume that these notices are inserted by the management of the hospital, and paid for out of the funds. If so, they are doubtless intended as a reminder to the living that they, too, are mortal, and that the National Hospital is a worthy object of charity: in other words, as a species of advertisement. Whether this unusual form of publicity produces results is in the nature of things difficult to guess. At the best it is a casting of bread upon the waters. Mr. Godfrey Hamilton could, no doubt, throw light upon the subject; and possibly the method is more efficacious than would at first sight seem likely. So long as one hospital alone pursues this particular policy the uniqueness of it must serve to attract attention, even if it does nothing more. But it can easily be seen that if the fashion spreads the *Times* will make more profit out of it than anyone else.

### IMPROVEMENTS AT COLNEY HATCH MENTAL HOSPITAL.

The alterations which the London County Council are about to carry out at Colney Hatch Mental Hospital provide for a new residence for the medical superintendent, the adaptation for patients of his present quarters, and the enlargement of the attendants' mess-room and a separate recreation room. The block hitherto occupied by the medical superintendent and the house steward will provide dormitory accommodation on the first and second floors for sixty-six male patients. The ground floor will be utilised for administrative purposes, including an assistant medical officer's bed and sitting rooms, a library and inspector's office, offices for head attendants, and a room in which tea for visitors can be provided. The new rooms for use as library and head attendant's office will release some adjacent rooms at present used for these purposes, the vacated rooms accommodating fifteen of the sixteen patients who will be displaced by the disuse of an unsatisfactory dormitory. Six single rooms in the main building will be used for the six extra attendants who will be required for the additional patients, and this will reduce the patients' beds by six, making the net addition to accommodation fifty-nine beds. The cost of the various alterations is estimated at about £4,000—the new residence of the medical superintendent £2,740, and the enlargement of the attendants' mess-room and the new recreation room £575.

### PROPOSED NEW NOTTINGHAM HOSPITAL.

The Nottingham Cripples' Guild is actively engaged in a work which may lead to the addition of more than one institution to the city. A school for physically defective children is hoped for under the control of the local authority, and the committee of the Guild is also urging the erection of a country hospital, where the children after operations for surgical tuberculosis can remain for a prolonged period of rest. The local education authority has been approached through its medical inspection sub-committee, and the city architect is reported to be engaged on preliminary plans. Before these can take effect, however, the Local Government Board and the education authority, to say nothing of the Guild, have to be satisfied.

### DR. JULIA COCK'S SUCCESSOR.

Miss Aldrich Blake, M.D., M.S., has been appointed to act temporarily as Dean of the London School of Medicine for Women, which is vacant owing to Dr. Julia Cock's death. Miss Blake is senior surgeon at the New Hospital for Women in Euston Road. A permanent appointment will probably be made in about a month.

### A QUESTION FOR SCHOOL MEDICAL OFFICERS.

School medical officers are well acquainted with the facts stated by Sir William Bennett at the annual dinner of the Illuminating Engineering Society last week. He pointed out once again that illumination is an important part of hygiene, and that light must be considered not merely from its intensity but from its non-irritant effects on eye and brain. The lighting of schools was an object-lesson: too little light led to eye-strain; too much, to irritation of eye and brain. In fact, bad lighting might lead in children to such defects as spinal curvature, and a large amount of brain-fag ascribed to overwork was really caused by badly arranged lighting. The number of school children wearing spectacles was on the increase, but there was no adequate inspection of the school illumination. He often wondered what the effect of such an inspection would be upon the number of spectacles required. This is a pregnant question for school medical officers, and we suggest that one or more sufficiently awake to the interest of the point should take it up and communicate the result of their investigations. Bad illumination is not confined to schools, however. Do we not all know instances of horribly lighted hospital wards?

### X-RAYS IN POOR-LAW INFIRMARIES.

The Lambeth Board of Guardians have received the sanction of the Local Government Board to instal the *x*-rays in their infirmary at Brook Street at an estimated cost of £207 without advertising for tenders. This will help to bring another Poor-Law infirmary up to date, and should be of great assistance to the medical staff. The installation of the apparatus has taken place in a large number of Metropolitan infirmaries, with valuable results for the sick poor, and it is to be hoped a further extension of the installation will take place in the near future in those institutions which at present do not possess it.

### PROPOSED REGISTER OF DISPENSERS.

The Bill which has been introduced in the House of Commons by Mr. W. S. Glyn-Jones "to provide for the registration and qualification of persons entitled to act as assistants to pharmacists in the compounding of prescriptions of duly qualified medical practitioners" is likely to contain proposals of considerable interest to institutional dispensers. The Bill has not yet been printed, but it will be remembered that its promoters, the Pharmaceutical Society, submitted the proposed terms of the measure to the General Medical Council last year. In that communication it was stated that it was thought desirable to embody in the Bill a proposal to set up a register of assistants, which should include, among others, persons who for three years prior to the passing of the Insurance Act had acted as dispensers to a public institution. If this proposal were embodied in an Act of Parliament dispensers would be accorded a definite status, and for that reason alone the proposal will be welcomed. Before discussing the matter more fully, however, it will be better to wait until the Bill is printed.

### THIS WEEK'S DRUG MARKET.

Business continues quiet, but there is a fair undercurrent of inquiry which will probably lead to business in due course. Price changes have not been numerous and the few that have taken place are not of special importance. The value of opium is not quite so firmly maintained, and morphine also has a slightly lower tendency. News from the Norwegian fishing-grounds is to the effect that operations are still interfered with by stormy weather; the catch of cod, however, shows signs of improvement, and offers of new season's cod-liver oil are made more freely, quotations being somewhat lower than the opening prices; it is somewhat difficult to decide whether it is advisable to buy at once or to delay making purchases until more progress has been made with the fishing, but the general attitude of buyers seems to indicate that a waiting policy is preferable. Cocaine still has a lower tendency in price, but, to repeat the advice recently given, it might be as well to replenish stocks at an early date. Menthol has a lower tendency in price. Essence of lemon is again cheaper, and the downward movement is likely to continue. Senega tends upwards in value and valerian rhizome is rather dearer.

### TO OUR READERS.

Contributions are specially invited from any of our readers to these columns. They should deal with topical subjects and news. They must be authenticated for the information of the Editor only. The minimum payment if published is 5s. There is no hard-and-fast rule as to space, but notes of about twenty lines in length are preferred.

